# Editorial: Acquisition of Clause Chaining

**DOI:** 10.3389/fpsyg.2020.583431

**Published:** 2020-10-30

**Authors:** Hannah S. Sarvasy, Soonja Choi

**Affiliations:** ^1^MARCS Institute for Brain, Behaviour and Development, Western Sydney University, Sydney, NSW, Australia; ^2^Department of Linguistics and Asian/Middle Eastern Languages, San Diego State University, San Diego, CA, United States

**Keywords:** clause chain, complex sentence, Japanese, Korean, Ku Waru, Turkish, Nungon, Pitjantjatjara

Research on the acquisition of complex syntax has largely overlooked a special type of complex sentence, found in hundreds of languages outside Western Europe: the clause chain. A clause chain contains as few as one and as many as 20 or more “medial” clauses, with verbal predicates that are under-specified for tense and other categories, and a single “final” (finite) clause, with a verbal predicate that is fully-specified for tense and, often, other categories. “Medial” clauses relate syntactically to other clauses in the chain without being subordinated to them. In some languages, each clause in a chain must indicate in advance whether the subject of the next clause will be the same as or different from that of the current clause, through “switch-reference” marking (Haiman and Munro, 1983; van Gijn and Hammond, 2016). Unlike English complex sentences, clause chains' distribution is partially predictable in that it is often associated with description of temporally sequential events or actions.

Clause chaining occurs in typologically diverse languages, but there has been no comprehensive cross-linguistic study of clause chaining; the comparative clause chain literature is limited to book chapters and working manuscripts (Longacre, 1985, 2007; Bickel, 2010; Dooley, 2010). This Research Topic presents the first-ever set of research articles focusing on or relating to children's acquisition of clause chains. Six of these articles describe and analyze child clause chain productions in languages in which clause chains are frequently used, especially to describe sequences of related events/actions/states. Of these, three focus on Eurasian languages—Korean (Choi), Japanese (Clancy), Turkish (Ögel-Balaban and Aksu-Koç)—and three focus on indigenous languages of Australia and New Guinea—Pitjantjatjara (Defina), Ku Waru (Rumsey et al.), and Nungon (Sarvasy). These six studies are analyzed in a seventh synthesis paper (Sarvasy and Choi). An eighth contribution describes and analyzes a complex sentence type in Sesotho that is similar to clause chains, but with some differences (Riedel et al.). Two final contributions provide fresh perspectives on acquisition of complex sentences in non-European languages without true clause chaining: Modern Hebrew (Berman and Lustigman), and K'iche' and Mam (Pye and Pfeiler).

The first six studies use diverse approaches. Defina, Rumsey et al., and Sarvasy present children's spontaneous production of clause chains from naturalistic, longitudinal studies in under-described languages: Pitjantjatjara of central Australia, and Ku Waru and Nungon of Papua New Guinea. Clancy and Ögel-Balaban and Aksu-Koç use cross-sectional data to investigate clause chain productions in Japanese and Turkish. Here, we note that Clancy is the first to use a mixed-effects statistical model to predict clause chain characteristics, such as chain length (in clauses) and when a speaker chooses to end a chain, for both adults and children. Choi presents both longitudinal and cross-sectional datasets and gives a synthetic analysis of clause chain acquisition in Korean children.

Despite these methodological differences, Sarvasy and Choi found that these studies present a coherent picture of early clause chain acquisition as involving: (a) morphologically error-free complex sentence production, (b) uniform progression from two-clause chains to longer chains, and (c) early accuracy in switch-reference marking and topic continuity in clause chains, albeit with marked cross-linguistic differences, based on distributions in the ambient languages.

Beyond the acquisition of clause chaining, the studies in this Research Topic contribute to a better understanding of the phenomenon of clause chaining in three important ways.

First, as yet, no one has pinned down criteria for differentiating between a “clause chaining language” and a “non-clause chaining language.” As Sarvasy points out, it is relatively easy to approximate a minimal clause chain in English with an adverbial clause-plus-main clause combination, but it is unnatural to stack three or more English adverbial clauses in one prosodic sentence—let alone 20! The acquisition data presented in this Research Topic may aid in differentiating between “non-clause chaining” languages like English, where such stacking of non-finite clauses is unnatural, and “clause chaining” languages like Japanese, Korean, Ku Waru, Nungon, Pitjantjatjara, and Turkish, where it is frequent and natural. Children acquiring “clause chaining” languages begin producing clause chains by around two-and-a-half years (Sarvasy and Choi). But Berman and Lustigman show that speakers of the “non-clause chaining language” Modern Hebrew only begin to produce sequences of non-finite clauses in their teens and older, in occasional use of an advanced, literary speech style. This contrasts with their early production of “extended predicate” multi-verb sequences within a single clause. Further, Sarvasy shows that children acquiring the Papuan language Nungon produce subordinate and coordinate sentences as well as clause chains before their third birthday, but that clause chains are produced with far higher frequency than the other two complex sentence types. (This remains to be confirmed for the other languages in this Research Topic.)

Second, these studies highlight the structural diversity of clause chains across languages. For instance, the number of distinct medial verb forms, with distinct semantic functions, ranges in these languages from just one (Pitjantjatjara, Nungon) to 100 (Korean). Further, the complex sentence types described by Riedel et al. for Sesotho are similar to clause chains in that they involve clauses with verbal inflections that are under-specified for tense, but different from typical clause chains in that the Sesotho forms can “skip” multiple intervening tensed clauses, often across multiple speakers. They thus represent an extra-long-distance type of clause linkage, the likes of which may never have been described before for any language.

Last, articles in this Research Topic have relevance for study of diachronic aspects of clause chaining. The minimal clause chain (comprising just one “medial” and one “final” clause) has structural counterparts in expressions employing an adverbial clause plus a main clause in many languages of the world (Bickel, 2010). Applying the comparative method of historical linguistics to child language (as in Pye, 2017), Pye and Pfeiler show that this sort of construction evolved into monoclausal complex predicates with morphological markers of directionality in different ways for the Mayan languages K'iche' and Mam. Indeed, more “grammaticalized” functions of clause chains—always two-clause chains—co-exist synchronically with non-grammaticalized clause chains in some of the other languages of the Research Topic, such as Japanese (Clancy), Ku Waru (Rumsey et al.) and Nungon (Sarvasy). If these languages follow the pattern of the Mayan languages, the Japanese, Ku Waru, and Nungon two-clause chain types with more grammatical functions could evolve into monoclausal complex predicates in the future.

The contributions to this Research Topic augment our understanding not only of the acquisition of this special type of complex sentence, but also of clause chain typology in general. We hope that this Research Topic will motivate further research on clause chaining in child language, as well as in adult grammar and language processing.

## Author Contributions

HS and SC both contributed to the writing of this paper.

## Conflict of Interest

The authors declare that the research was conducted in the absence of any commercial or financial relationships that could be construed as potential conflicts of interest.
